# Effect of ROI filtering in 3D cone‐beam rotational angiography on organ dose and effective dose in cerebral investigations

**DOI:** 10.1120/jacmp.v16i2.5306

**Published:** 2015-03-08

**Authors:** Fabian Göpfert, Ralph Schmidt, Jörg Wulff, Klemens Zink

**Affiliations:** ^1^ Institut für Medizinische Physik und Strahlenschutz, Technische Hochschule Mittelhessen Gießen Germany; ^2^ Klinik für Strahlentherapie und Radioonkologie, Universitätsklinikum Gießen‐Marburg Germany

**Keywords:** region‐of‐interest imaging, cone‐beam rotational angiography, GMctdospp, Monte Carlo simulation, EGSnrc

## Abstract

The assessment of intracranial aneurysms is increasingly performed using three‐dimensional cone‐beam rotational angiography (3D CBRA). To reduce the dose to the patient during 3D CBRA procedures, filtered region‐of‐interest imaging (FROI) is presented in literature to be an effective technique as the dose in regions of low interest is reduced, while high image quality is preserved in the ROI. The purpose of this study was to quantify the benefit of FROI imaging during a typical 3D CBRA procedure in a patient's head region. A cone‐beam rotational angiography unit (Infinix) was modeled in GMctdospp, an EGSnrc‐based Monte Carlo software, which calculates patient dose distributions in rotational computed tomography. Kodak Lanex, a gadolinium compound, was chosen to be the ROI filter material. The adult female ICRP reference phantom was integrated in GMctdospp to calculate organ and effective doses in simulations of FROI‐CBRA examinations. During the Monte Carlo simulations, different parameters as the ROI filter thickness, the ROI opening size, the tube voltage, and the isocenter position were varied. The results showed that the reduction in dose clearly depends on these parameters. Comparing the reduction in organ dose in standard 3D CBRA and FROI‐CBRA, a maximum reduction of about 60%–80% could be achieved with a small sized ROI filter and about 40%–70% of the dose could be saved using a ROI filter with a large opening. Further we could show that dose reduction strongly depends on filter thickness, the location of the organ in the radiated area, and the position of the isocenter. As a consequence, dose reduction partially differs from theoretically calculated values by a factor up to 1.6. The effective dose could be reduced to a minimum of about 40%. Due to the fact that standard 3D CBRA is only used for the assessment of aneurysms at present and, thus, most of the patient dose originates from the aneurysm treatment (with 2D techniques) itself, the dose reduction effect of ROI filtering in 3D CBRA tends to be much smaller, if the patient dose of a whole aneurysm treatment procedure is considered.

PACS numbers: 87.59.DJ, 87.55.kh

## I. INTRODUCTION

Three‐dimensional cone‐beam rotational angiography (3D CBRA) is frequently used in modern neuroradiology, as it facilitates diagnosis and planning treatment of intracranial aneurysms.^(1)^ Compared with conventional two‐dimensional (2D) digital subtraction angiography, 3D CBRA provides more detailed information for the assessment of cerebral aneurysms and allows more exact depiction of anatomic details that are beneficial for interventional surgery.^2^, ^3^, ^4^, ^5^, ^6^ Regarding the effective dose to the patient Bridcut et al.^(7)^ reported a mean total effective dose of 3.4 mSv for the assessment of aneurysms in a conventional coiling procedure. Using 3D CBRA instead of 2D techniques could reduce the dose to about 0.2 mSv, which is due to the reduced tube current per frame in 3D CBRA.^(7)^

Even so, patient dose from 3D CBRA is significantly lower than that for 2D DSA series used in the evaluation process, a variety of technical approaches to further reduce the patient dose in 3D CBRA are described in literature^(8)^ following the ALARA principle ALARA=“as low as reasonably achievable”.

Apart from tube current modulation or peak kilovoltage optimization, which is by now good practice in rotational cone‐beam CTs, a number of authors investigated the feasibility of filtered region of interest (ROI) imaging.^9^, ^10^, ^11^ In many procedures the region of interest, such as the area of the intervention, the location of the aneurysm or the position of stents and coils, is considerably smaller than the detectors field of view (FOV). The X‐ray beam could be collimated to the form of the ROI. On the one hand a remarkable dose reduction can be achieved. On the other hand artifacts occur in the reconstruction process as soon as the adjusted FOV does not include the entire body structure in each projection. For this reason filtered ROI (FROI) imaging was introduced to attenuate the X‐ray beam substantially (but not completely) to preserve sufficient attenuation data for 3D image reconstruction.

In FROI imaging, an additional X‐ray attenuating filter with an aperture is placed in the beam path to reduce the exposure only outside the ROI, keeping the desired image information. Schafer et al.^(1)^ developed a procedure that allows an artifact‐free 3D image reconstruction of the filtered image data. In their research they used Kodak Lanex, a gadolinium compound (Kodak, Rochester, NY), as the ROI filter material. Depending on the filter thickness and the ROI size, Schafer et al. evaluated the potential dose savings, both theoretically and experimentally, characterized by the dose area product. The results showed a considerable dose decrease. For instance, a filter thickness of 1.29 mm and a ROI size of 20% (40%) of FOV lead to a reduction in dose of about 75% (60%).

However, the impact of FROI imaging on patient organ doses, as well as the effective patient dose, was not investigated by Schafer et al.^(1)^ Thus the aim of this study is to evaluate the dose reduction effect on organ doses in a patients head area. For this purpose, we performed Monte Carlo simulations using the adult female reference phantom of the ICRP^(12)^ in conjunction with the EGSnrc^(13)^‐based Monte Carlo tool GMctdospp.^14^, ^15^, ^16^ Therefore, a representative source model of a 3D CBRA scanner was developed.

## II. MATERIALS AND METHODS

### A. Monte Carlo method

To determine organ doses and effective patient dose in CT examinations, the software GMctdospp was used. GMctdospp represents a graphical user interface (GUI) for the Monte Carlo radiation transport package EGSnrc extended by a specific user code which allows the rotation of the source, including filter geometries, in the beam path such as the prefilter, as well as the ROI filter. In all simulations, secondary electron transport was disabled by setting the cutoff energy for electrons (global ECUT) to 0.2 MeV. This means, kerma equals dose, which is a valid approximation for patient‐equivalent tissue and in the energy range of diagnostic imaging.^(17)^ Photons were simulated down to 1 keV (global PCUT). Beside activated Rayleigh scattering defaults were kept for all other EGSnrc input parameters. The number of initial particles was 10^8^ for all phantom studies. Following a simplified approach from Caon et al.^(18)^ and Jones and Shrimpton,^(19)^ only the weighted attenuation of the beam passing the filter materials is calculated, as scattering in the filter materials is negligible for the following dose calculations. Applying this technique to Monte Carlo CT codes has already been shown to be consistent (e.g., by Jarry et al.^(20)^ or Long et al.^(21)^).

### B. ROI filter modeling

The Monte Carlo model of the region of interest filter was adapted to the measurements of Schafer et al.,^(1)^ who used four different thicknesses (layers) for the ROI filter: 0.43 mm, 0.86 mm, 1.29 mm, and 1.82 mm. The filter material was Kodak Lanex, a mixture of gadoliniumoxysulfide and plastic materials that is commercially available. To simplify the simulations, pure gadoliniumoxysulfide, which is responsible for the main attenuation, was chosen as ROI filter material.

First, to obtain identical transmission properties a source model of the Toshiba Infinix angiography system (Toshiba Medical Systems Corp., Chicago, IL) used in the measurement performed by Schafer et al.^(1)^ was created. Necessary X‐ray spectra (80 kVp (kVp=kilo voltage peak), 90 kVp, and 100 kVp) were generated by an algorithm of Boone and Seibert.^(22)^ Apart from the ROI filter material, a prefilter according to manufacturer information (0.3 mm copper and 2.6 mm aluminum) was included in the model to ensure veridical simulation conditions.

Second, to define an effective thickness of the filter material, the transmission of pure gadoliniumoxysulfide was simulated, increasing the thickness of the modeled filter iteratively until simulated and measured relative transmission matched best.

In the next step the EGSnrc geometries of the ROI filters were generated as rectangular shapes with circular apertures in the size of 20% of FOV and 40% of FOV. The detector's FOV was $20\times 20\;{\text{cm}}^{2}$.

### C. Patient geometry and table modeling

The adult female ICRP reference phantom^(12)^ was chosen to calculate organ doses in simulations of 3D CBRA examinations. The anthropomorphic phantom is based on a real patient study, and includes complete segmentation and material definitions of tissues and organs at risk. To estimate the influence of a patient table, a Visual‐Basic tool was developed to extend the voxel matrix with a rectangular shape representing the pillar.

In order to model the patient table correctly, transmission measurements at different tube voltages were necessary. Bednarek et al.^(23)^ measured the transmission of the Toshiba Infinix System, but solely at a single tube voltage of 80 kVp. As the Toshiba Infinix system was not available at the time of table modeling, we decided to collect measurement data from a Siemens Artis zee biplane system (Siemens Medical Solutions, Inc., Forcheim, Germany), as the attenuation effect of this patient table was assumed to be in the same range as the attenuation of the Toshiba system. To approve this, we compared the calculated attenuation of our modeled patient table and Toshibas head model at 80 kVp.

As there are no exact data from the manufacturer about the material composition, we decided to evaluate an effective density instead of an effective thickness to obtain identical attenuation properties. Pure carbon was chosen as simulation material, as it is a typical material component in patient tables.^(24)^ To adapt the density in Monte Carlo simulations, attenuation measurements of the patient table were performed in a real Siemens CT system at all adjustable tube voltages: 70 kVp, 81 kVp, 90 kVp, 96 kVp, and 102 kVp, using a solid state detector (RaySafe Xi R/F detector, UnforsRaySafe AB, Billdall, Sweden). During the measurements tube current modulation was disabled. Consecutively, the effective density was defined in Monte Carlo simulations by iteratively increasing the density of the patient table until simulated and measured relative transmission matched best. A total prefiltration of 2.5 mm aluminum was applied. The necessary X‐ray spectra were generated, as described in the Materials & Methods section B.

### D. Determination of organ doses using the ICRP reference phantom

In order to obtain absolute organ doses in GMctdospp, a calibration factor CF was defined to achieve the relationship between simulated relative dose ${\left(D_{air}/\phi \right)}_{MC}$ in Gy/primary photon fluence ϕ and measured absolute dose ${\left(D_{air}/I\cdot t\right)}_{meas}$ in Gy/mAs. This concept is based on Jarry et al.^(20)^ and DeMarco et al.^(25)^

(1) $$CF=\frac{{\left(D_{air}/I\cdot t\right)}_{meas}}{{\left(D_{air}/\phi \right)}_{MC}}$$

$D_{air}$ was taken from a dose measurement in air by Schafer et al.^(1)^ with the following parameters: tube current I=250 mA, exposure time t=6.3 ms, source–imager distance: 110 cm, source–surface distance: 70 cm, 106 projections, 2° spacing, starting angle: 106°. The ionization chamber was placed free in‐air at the isocenter to determine air kerma during source rotation. Applying the identical adjustment of the virtual cone‐beam CT, a calibration simulation was carried out to obtain $D_{air}/\phi$. Calibration factors were determined for two tube voltages, 90 kVp and 100 kVp. Following the results of Gregory et al.^(26)^ the effect of tube current modulation (TCM) was neglected, as the human head has a quite symmetric form and density distribution. Moreover, neglecting TCM serves as a conservative approach of estimating organ dose and effective dose to the patient.

**Figure 1:**
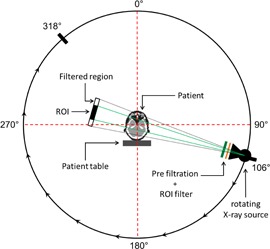
Simulation setup including the X‐ray source, inherent filtration, ROI filtration, ICRP female phantom, and the patient table. The X‐ray source is performing a rotation in the angle range from 106° degree to 318°.

**Figure 2:**
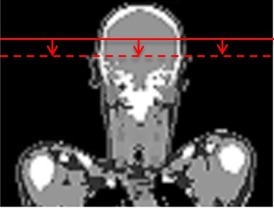
30 mm shift of the isocenter Z position during the Monte Carlo simulations in order to investigate the impact of isocenter variance in clinical practice. First the isocenter coordinates were set to X: 265 mm, Y: 90 mm and Z: 1580 mm in the female ICRP reference phantom.^(12)^ In a second Monte Carlo simulation, the isocenter was set to X: 265 mm, Y: 90 mm and Z: 1550 mm.

The modified voxel matrix of the adult female ICRP reference phantom was integrated in GMctdospp using the verified ROI filter geometries, different ROI filter thicknesses, filter types (ROI opening size), and tube voltages. Monte Carlo simulations were performed in the patient's head region. Figure 1 demonstrates the simulation setup. First, the isocenter was set according to an image taken from a real CBRA investigation from Schafer et al.^(1)^ (coordinates of the isocenter in the voxel matrix of ICRP phantom: X: 265 mm, Y: 90 mm, Z: 1580 mm). In a second run, the isocenter Z position was set 30 mm further down (X: 265 mm, Y: 90 mm, Z: 1550 mm), as shown in Fig. 2. This was done to investigate the effect of different isocenter positions, as in clinical practice the isocenter location is variable.

In order to investigate the influence of the patient table, the original voxel matrix of the adult female ICRP reference phantom was integrated in GMctdospp and the same simulations with the same parameter variations as described above were repeated.

The organ doses $H_{T}$ of the spinal cord, brain, left/right salivary glands, and left/right eye lens were calculated following the approach of Jarry et al.^(20)^ and DeMarco et al. ^(25)^:

(2) $$D_{i,absolute}=D_{i,simulated}\cdot CF\cdot I\cdot t$$

(3) $$H_{T}=w_{r}\cdot \frac{\sum_{i\in organ}D_{i,absolute}\cdot \rho_{i}\cdot \upsilon_{i}}{\sum_{i\in organ}\rho_{i}\cdot \upsilon_{i}}$$

Here, $D_{i}$ describes the dose to a voxel *i* in the simulated voxel matrix, $\rho_{i}$, is the density of a voxel with volume ν_*i*_. According to ICRP 103,(27) the equivalent dose $H_{T}$ can be calculated by multiplying the radiation weighting factor $w_{r}$ with the mean absorbed dose within the organ. The effective dose *E* is defined as

(4) $$E=\sum_{T}w_{T}\cdot H_{T}$$

where $w_{T}$ is the related tissue weighting factor for each organ considered. In our study the effective dose was calculated using the tissue weighting factors for bone marrow, bone surface, salivary glands, thyroid, esophagus, brain, lung, skin, and remaining structures according to ICRP 103,^(27)^ as these organs at risk received a relevant amount of dose.

Although the dose to the eye lens does not contribute to the effective patient dose, it is considered in particular as it is quite radiosensitive due to deterministic effects.^28^, ^29^

## III. RESULTS & DISCUSSION

### A. Effective thickness of ROI filter and patient table verification

Table 1 shows the calculated effective thickness of pure gadoliniumoxysulfide used as ROI filter material in the Monte Carlo simulations for different tube voltages. The statistical uncertainty (type A uncertainty^(30)^) of all Monte Carlo simulations in the ROI filter adaption was below 0.2%. Due to a small variation of the effective thickness at different tube voltages, average thicknesses (0.102 mm, 0.204 mm, 0.306 mm, 0.408 mm) were determined by taking the mean of all calculated effective thicknesses. This was done to obtain a uniform thickness for each layer. The maximum deviation between simulated effective thicknesses and the average effective thickness across all layers and kVp values was 5.4%, which is within the framework of the present study.

The results of the determination of an effective density for the patient table's headrest are presented in Table 2. A mean effective carbon density of 0.484 g/cm³ was calculated and applied in subsequent Monte Carlo simulations for the ICRP phantom. The maximum deviation between the effective carbon density determined for the respective tube voltage and the average effective density was below 2% (see Table 2). A further Monte Carlo investigation simulating the modeled Siemens table with the Toshiba beam quality showed that the transmission at 80 kVp tube voltage is 0.77 and, therefore, is slightly below the transmission measured by Bednarek et al.^(23)^ Monte Carlo studies simulating the ICRP phantom matrix with and without integrated patient table leads to a difference in organ and effective does of less than 8%. Obviously, the influence of patient table on organ dose and effective dose is low. Hence, slight variations in table attenuation are negligible.

**Table 1:** Monte Carlo based calculation of the effective ROI filter thicknesses (pure gadoliniumoxysulfide) compared to the real filter thicknesses (1–4 layers of Kodak Lanex) used in Schafer et al.^(1)^ The statistical uncertainty (type A uncertainty^(30)^) was below 0.2%

*Real Filter Thickness (Schafer et al.* ^(1)^) *(mm)*	*80 kVp Effective Thickness (mm)* ^a^	*90 kVp Effective Thickness (mm)* ^a^	*100 kVp Effective Thickness (mm)* ^a^	*Average Effective Thickness ± 2*σ *(mm)*
0.43	0.107 (5.4)	0.105 (2.7)	0.104 (1.8)	0.102±0.006
0.86	0.208 (2.1)	0.204 (0.2)	0.203 (0.5)	0.204±0.011
1.29	0.306 (0.1)	0.309 (1.0)	0.298 (2.4)	0.306±0.017
1.73	0.399 (2.0)	0.391 (4.1)	0.391 (4.1)	0.408±0.022

^a^ The number in brackets shows the deviation to the average in %.

**Table 2:** Determination of the effective density of the patient table's headrest in Monte Carlo simulations. The statistical uncertainty (type A uncertainty^(30)^) was below 0.2%

*Tube Voltage (kVp)*	*Measured Transmission*	*Effective Carbon Density (g/cm³)*	*Deviation to Average (%)*
70	0.683	0.475	1.9
81	0.687	0.491	1.3
90	0.695	0.491	1.3
96	0.701	0.488	0.9
102	0.709	0.478	1.4

### B. Organ dose and effective dose in FROI‐CBRA

**Figure 3:**
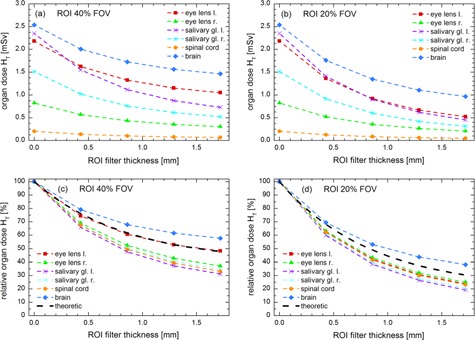
Absolute and relative organ doses determined for 3D CBRA in Monte Carlo simulations of the female reference phantom as a function of the ROI filter thickness. The tube voltage was set to 100 kVp and the isocenter Z position was adjusted to 1580 mm (see Fig. 2). (a) Absolute organ doses, ROI size=40% FOV; (b) absolute organ doses, ROI size=20% FOV; (c) relative organ doses, ROI size=40% FOV; (d) relative organ doses, ROI size=20% FOV.

Figure 3 shows the absolute and the relative dose saving effect of ROI filtering in a typical 3D CBRA investigation (100 kVp tube voltage, Z position: 1580 mm) of the patient's head region. Without any ROI filtering, an organ dose of about 2.5 mSv was calculated for the brain, while the spinal cord received the lowest dose of about 0.25 mSv. As expected, if a ROI filter is inserted into the X‐ray beam, the dose decreases in an exponential fashion with increasing filter thickness. For instance, regarding the left (right) salivary gland, an initial organ dose of 2.3 mSv (1.5 mSv) can be reduced to 0.75 mSv (0.5 mSv) applying a ROI filter with 1.72 mm thickness and an opening size of 40% FOV (Fig. 3(a)). This corresponds to a relative reduction of 30% (35%), as shown in Fig. 3(c).

Moreover, the impact of ROI filtering depends on the ROI size. As expected, the dose reduction was more effective with the small‐sized ROI filter (20% FOV) (Figs. 3(b) and (d)) compared to the large one (40% FOV, see Figs. 3(a) and (c)). Consider, for example, the organ dose in brain, a large ROI filter with a thickness of 0.43 mm reduces the dose around 20%, while the same filter with a small opening saves about 30%.

Schafer et al.^(1)^ calculated the potential dose savings of ROI filtering theoretically regarding the dose area product. In Figs. 3(c) and (d) the theoretical dose reduction of ROI filtering according to Schafer and colleagues is presented. In comparison to the theoretical consideration, the average dose saving for the brain was significantly lower (e.g., about 9% for the big sized ROI filter and 1.5 mm ROI filter thickness), whereas for the remaining organ structures the reduction in dose is larger (e.g., about 10% for the big sized ROI filter and 1.5 mm ROI filter thickness). This is due to the position of the different organs in the radiation field. Several organs are located either completely or with a large part of their volume in the peripheral, attenuated region. As a consequence, dose reduction is minted. In contrast, other organs like the brain (Figs. 3(c) and (d)) or the left eye lens (Fig. 3(c)) are influenced more intensively by the ROI and consequently receive higher doses. Hence, regardless of the gadolinium filter thickness, using a small‐sized ROI filter instead of a large one can cause a huge reduction in dose. These results also indicate that the theoretical consideration of relative dose savings in 3D CBRA investigations, as presented in Schafer et al.,^(1)^ may not be sufficient and only serves as an approximation.

**Figure 4:**
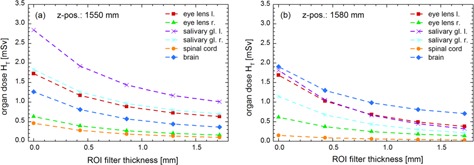
Absolute organ doses determined by Monte Carlo simulations of the female reference phantom (head area) as a function of the ROI filter thickness. The tube voltage was set to 90 kVp and the small‐sized ROI filter (ROI 20% FOV) was inserted. (a) Absolute organ doses, Z position=1550 mm; (b) absolute organ doses, Z position=1580 mm.

Figures 4 (a) and (b) show the organ doses simulated with 90 kVp tube voltage and a small‐sized ROI filter, but different isocenter Z positions in the reference phantom (1550 mm vs. 1580 mm). Comparing the organ doses, a conspicuous variation in dose can be determined for the salivary glands, the brain, and the spinal cord, while the organ dose in both left and right eye lens remains unchanged. The further up the isocenter is defined in the patients head area along the patient's z‐axis, the higher the organ dose is for the brain and the lower the doses turn out in the salivary glands or the spinal cord. The right eye lens is located in the attenuated area of the ROI filter. Thus the organ dose stays rather unaffected, if the isocenter Z position is changed. On the other hand, the dose to the left eye lens is unchanged due to the fact that the X‐ray source does not perform a complete 360° rotation and, therefore, the eye lens is not exposed directly. As a result, the dose to the left eye lens is influenced only marginally by changing the isocenter Z position.

**Figure 5:**
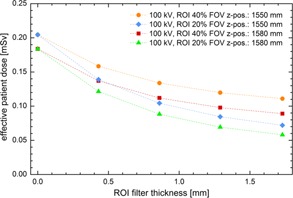
Plot of absolute effective dose determined in Monte Carlo simulations of the female reference phantom (head area) as a function of the ROI filter thickness. Different ROI sizes (ROI 20% FOV, ROI 40% FOV) and isocenter positions (Z position=1550 mm, Z position=1580 mm).

Figure 5 shows the effective dose administered to the patient in a 3D CBRA investigation procedure as a function of the ROI opening size and the isocenter Z position. The 100 kVp X‐ray spectrum was chosen. Without inserting a ROI filter into the X‐ray beam, an effective dose of about 0.18 mSv was calculated for an isocenter Z position of 1580 mm, whereas about 0.2 mSv were determined, if the isocenter is located at 1550 mm. Obviously, the further down the isocenter is defined along the patient's z‐axis the more organs, which are sensitive to radiation (for example, the salivary glands), are irradiated. Considering the dose reduction effect, the use of a large sized ROI filter with a thickness of 0.86 mm reduces the effective dose for example to 0.11 mSv (Z position: 1580 mm) and 0.13 mSv, respectively (Z position: 1550 mm). Using a filter with a small ROI opening and a thickness of 1.72 mm, the effective dose can be decreased to a value of 0.058 mSv (Z position: 1580 mm) and 0.072 mSv, respectively (Z position: 1550 mm). This corresponds to a relative dose reduction of 69%, and 65%, respectively.

It should be noted that although tube current modulation was not simulated, the calculated values in Fig. 5 agree well with the phantom measurements from Bridcut et al.,^(7)^ as well as from Gosch et al.^(31)^ The good agreement confirms the approach of Gregory et al.^(26)^ and shows that neglecting tube current modulation in Monte Carlo simulations of head CT examinations is consistent, and that the calculation of absolute organ/effective doses leads to reliable dose values.

Considering the effective dose reduction, Bridcut et al.^(7)^ report an average effective dose of about 13 mSv for the whole procedure for cerebral aneurysms with a 2D technique. About 3.4 mSv (about 26%) of these 13 mSv can be attributed to the assessment process of the aneurysm, on average. In comparison to this technique, applying 3D CBRA in the assessment stage may reduce the dose to the patient to a mean value of 0.2 mSv, which corresponds to a relative dose reduction of 94% considering the assessment and 25% if the whole investigation is considered. As shown in this study, inserting a ROI filter into the X‐ray beam during 3D CBRA could further reduce the relative dose to a minimum of more than 98% for the assessment, respectively nearly 26% for the whole procedure.

Compared to normal standard 3D CBRA, the increase in dose reduction (about 1%) due to ROI filtering in 3D CBRA is only marginal if the effective dose during a whole investigation is considered.

While Bridcut et al.^(7)^ report 13 mSv for the whole treatment process, other authors in literature report median effective doses in the range of 1.7 mSv to 36 mSv,^32^, ^33^ which is due to different complexity of the procedures, different imaging setups and protocols, as well as varying experiences of the radiologist. Assuming that the assessment procedure takes always about a quarter of the effective patient dose in a complete conventional procedure, the implementation of FROI imaging in CBRA leads to higher relative dose reductions in high‐dose procedures, and respectively lower relative dose reductions in investigations with low effective patient doses.

Considering the dose to the eye lens, the ICRP recommends in its 2011 statement^(28)^ to reduce the equivalent dose limit for the eye lens, as there are different evidences from epidemiological studies that any threshold for radiation‐induced cataracts is much lower than the originally expected value of 2 Gy acute exposure recommended in 2007.^(27)^ The evaluation of the epidemiologic data led to the assumption that a low threshold of 0.5 Gy — or even no threshold — is appropriate. However, considering the dose in a whole cerebral angiography investigation using 2D technique, Sandborg et al.^(34)^ reported an average dose to the eye lens of 140–200 mSv. Regarding the dose to the eye lens in 3D CBRA at 100 kVp (Fig. 3(a)), about 2.2 mSv were determined without ROI technique, which could be reduced to nearly 0.5 mSv if a small‐sized ROI filter with a thickness of 1.72 mm is inserted into the X‐ray beam. As a consequence, using FROI‐CBRA in the assessment stage of an interventional procedure and assuming that the assessment procedure takes always about a quarter of the patient dose in a whole conventional procedure, the eye dose could be reduced by about 25%. Adapting the dose range from 140–200 mSv presented by Sandborg and colleagues, the eye dose could be reduced to an average value about 105–150 mSv.

## IV. CONCLUSIONS

In this study we quantitatively evaluated the potential dose savings of region‐of‐interest imaging applied to a typical neuroradiological investigation. The results showed that a relative reduction in organ dose up to 80% compared to the dose preserved in standard CBRA without ROI filtering can be achieved. Thereby the dose reduction strongly depends on the ROI opening size and the thickness of the gadolinium filter, as well as the location of the organ, respectively the isocenter position. The effective dose to the patient could be reduced to a minimum of 40% compared to standard CBRA. We also demonstrated that a solely theoretical consideration of dose saving in ROI filtering as performed by Schafer et al.^(1)^ is only an initial estimate and only serves as an approximation, as dose reduction partially varied from theoretically calculated values by a factor up to about 1.6 (see Fig. 3(c)).

At present, CBRA is only used in the assessment stage of treating intracranial aneurysms in neuroradiology. Using standard CBRA without ROI filtering in the assessment stage instead of 2D imaging techniques, about 94% of the dose could be saved. The dose reduction could further increased to about 98% if FROI‐CBRA is applied for assessment.

Considering the dose to the patient during a treatment procedure, about 26% of the whole dose stems from assessment if 2D technique is used. Applying the dose reductions found in this study due to standard CBRA without ROI filtering (about 94%) and FROI‐CBRA (about 98%), about 25% of the overall dose could be saved using standard CBRA and nearly 26% could be saved with FROI‐CBRA. Obviously the gain in dose reduction using FROI‐CBRA instead of standard CBRA is low, if the whole treatment procedure is considered.

Hence, we advocate the application of 3D CBRA in the assessment of aneurysms in cerebral investigations, but we do not recommend making huge efforts using FROI‐CBRA. Another reason against using the ROI filter technique is the loss in image quality in the filtered region, as discussed in detail in Schafer et al.^(1)^ Consequently, since the location of the target region is not always known exactly, the risk of incorrect exposes exists.

## ACKNOWLEDGMENTS

The authors would like to thank Sebastian Schaefer and colleagues for providing their measurement data. Further thanks to the Department of Radiology from the University Medical Center Giessen for providing its angiography system.
